# An international review of tobacco smoking in the medical profession: 1974–2004

**DOI:** 10.1186/1471-2458-7-115

**Published:** 2007-06-20

**Authors:** Derek R Smith, Peter A Leggat

**Affiliations:** 1 International Centre for Research Promotion and Informatics, National Institute of Occupational Safety and Health, Kawasaki, Japan; 2 Anton Breinl Centre for Public Health and Tropical Medicine, James Cook University, Townsville, Australia

## Abstract

**Background:**

Tobacco smoking by physicians represents a contentious issue in public health, and regardless of what country it originates from, the need for accurate, historical data is paramount. As such, this article provides an international comparison of all modern literature describing the tobacco smoking habits of contemporary physicians.

**Methods:**

A keyword search of appropriate MeSH terms was initially undertaken to identify relevant material, after which the reference lists of manuscripts were also examined to locate further publications.

**Results:**

A total of 81 English-language studies published in the past 30 years met the inclusion criteria. Two distinct trends were evident. Firstly, most developed countries have shown a steady decline in physicians' smoking rates during recent years. On the other hand, physicians in some developed countries and newly-developing regions still appear to be smoking at high rates. The lowest smoking prevalence rates were consistently documented in the United States, Australia and the United Kingdom. Comparison with other health professionals suggests that fewer physicians smoke when compared to nurses, and sometimes less often than dentists.

**Conclusion:**

Overall, this review suggests that while physicians' smoking habits appear to vary from region to region, they are not uniformly low when viewed from an international perspective. It is important that smoking in the medical profession declines in future years, so that physicians can remain at the forefront of anti-smoking programs and lead the way as public health exemplars in the 21^st ^century.

## Background

Smoking represents a critical international issue for public health policy makers and strategists. According to the World Health Organisation, tobacco is the second major cause of death and the fourth most common risk factor for disease, worldwide. If current trends continue it will be causing around 10 million deaths each year by 2020, with approximately 650 million fatalities overall [1]. Smoking also represents a key issue in the medical profession, as physicians play a leading role in tobacco usage prevention in the community [2], and a key position in the development of overall public health policy. Medical professionals are on the frontlines of primary health care, and research has shown that medical interventions can be effective in helping patients to quit smoking [3]. In this role, physicians are widely viewed as exemplars by the community, their patients and their colleagues. Indeed, the physicians' office and hospital should be a model of non-smoking behaviour [4], and, as early as 1976, it was suggested that physicians could best persuade patients to quit if they themselves did not smoke [5].

Aside from its significant impact on patients' health, tobacco usage also represents an important occupational health issue in the medical profession. According to the International Labour Office (ILO), the promotion of smoke-free environments forms a key part of any healthy and safe workplace [6]. Interestingly, some of the first epidemiological research demonstrating the adverse health effects of tobacco smoking was actually conducted among a cohort of British physicians [7]. So important was Doll and Hill's 1954 study of British doctors that it was republished by the British Medical Journal 50 years later [8] and remains a milestone in public health to this day [9-11]. Further research from the United States (US) also supported the preliminary British findings with regard to smoking hazards [12-14].

Although the dangers of smoking are now well-known throughout the medical profession, physicians have not always set a good example for patients [15]. In the 20^th ^century for example, some physicians even *advertised *cigarettes [16,17]. Smoking rates among them were also quite high. Some of the earliest large-scale epidemiological research from the United States revealed that around 40% of physicians were smokers in 1959 [5], a figure which had fallen to 21% by the mid 1970s [18,19]. By the mid 1980s, around 17% of US physicians were still smoking cigarettes and 8% smoking pipes or cigars [20]. A large prospective study undertaken by the American Cancer Society in 1982 revealed a smoking prevalence rate of around 25% among physicians [21]. Subsequent National Health Interview Surveys found that the national smoking rate for physicians in the US had fallen dramatically between 1987 and 1994, and was below 10% by the mid 1990s [22-24]. Similar downward trends were also seen in Scandinavia [25] and the Netherlands [26,27] during the latter half of last century.

Although these investigations suggest that physicians' smoking rates are probably declining in many parts of the world, international trends have not been clearly elucidated. Furthermore, few, if any, researchers have comprehensively reviewed tobacco-smoking habits in the medical profession from a global perspective. This represents an unfortunate oversight in the literature, as data on physicians' smoking habits has at least two direct benefits for health care policy makers [15]. Firstly, it can help predict how effective any potential anti-smoking campaigns in the wider community might actually be. Secondly, and perhaps most importantly, contemporary tobacco usage data allows public health policy makers to determine how soon their overall community prevalence rate might decline [15]. Given such clear benefits, the aim of our investigation therefore, was to undertake a comprehensive review of international tobacco smoking surveys, which have been conducted among physicians over the past 30 years. The main research question was, what proportion of physicians are smoking in what countries, and how have their habits changed over time.

## Methods

This study was conducted as an extensive international review targeting all manuscripts published in peer-reviewed journals relating to the topic of tobacco smoking among physicians. No unpublished articles were included. As the nature of research changes over time and results quickly go out of date, only manuscripts which had been published in the previous 30 years were included. As there is always some delay between conducting a study and actually having it published, the most recent investigations on this topic had been conducted in 2004, and thus the search criteria were limited to articles published between 1974 and 2004. For consistency, only English-language reports were included in the review. The literature review began in March 2006 with a Medline and CINAHL (Cumulative Index to Nursing and Allied Health Literature) internet search using the most appropriate Medical Subject Headings (MeSH) 'smoking', 'tobacco' and 'physician'. After identifying some initial studies, the search was repeated using the additional keyword variations of 'smoke' and 'doctor'. Manuscripts located using these initial criteria were subsequently examined to find additional publications in their reference list. A large proportion of manuscripts were eventually located using the latter method. Manuscripts were arranged in descending order, depending on the year in which the survey was undertaken, rather than the publication year. Where such information was unavailable, the corresponding author of the manuscript was contacted for clarification. In cases where contact with the authors was not possible or repeatedly unsuccessful, manuscripts were listed by year of publication and marked with an asterisk. If a study had been undertaken over the course of more than one year, then the most recent year was listed.

Manuscripts were assigned a reference number based on the abovementioned criteria. As the results of some investigations were published in more than one journal article, some studies have two to three corresponding references. Smoking rates were listed as the prevalence of smoking among the entire group, and also as prevalence rates for males and females. In cases where smoking prevalence rates by gender were not stated in the manuscript itself, they were manually calculated whenever possible. For consistency, all smoking prevalence rates were rounded to the nearest whole number. Response rates for each study were also rounded to the nearest whole number for standardisation purposes. As some studies investigated multiple occupational groups that included physicians, some response rates were indicative of the entire group response, rather than just the physicians. Where authors had apparently used a convenience sample with an unspecified response rate, or the response rate was not listed, this information was also indicated on the table.

## Results

A total of 81 published studies met the inclusion criteria [28-113], as shown in Table 1. Most (n = 48) had been conducted as postal surveys, 14 were hand delivered, 4 were telephone surveys, 4 had utilised census data, 3 were conducted by personal interview, and two had been conducted at conferences. A further 4 used both postal surveys and follow-up telephone interviews, while two used postal and hand delivery, mainly to increase response rates following the postal phase. The latter technique appears to have been quite successful in some cases, with one Malaysian study [76] achieving a 100% response rate in this regard. Response rates of the published studies ranged from 27% [101,102] to 100% [76], with most above 60%. Only four manuscripts had response rates below 50%, and three investigations did not list their response rate. One study from Iran appeared to have 100% participation [39], although a response rate was not clearly stated and the authors were unable to be contacted to clarify their result, despite repeated attempts. A similar situation was encountered with a Greek study [70], where no response rate was listed and the authors were unable to be contacted. Among all manuscripts included in the current review, sample sizes ranged from 45 [29] to 10 807 [77], with an encouraging proportion having over 1000 respondents. Particularly large surveys of physicians' tobacco smoking habits were published from the Doctor's Health Study in the United Kingdom [77], the Physicians Health Study in the United States [66-68], and also from New Zealand census data [54,106,111]; one of the few countries in the world which includes tobacco smoking questions on their census form [54].

**Table 1:** International Comparison of Tobacco Smoking Surveys Conducted among Physicians between 1974 and 2004

**Publication Details**			**Smoking Rate**^c^			**Study Details**			
**Authors **^a^	**Year **^b^	**Country**	**All**	**Male**	**Female**	**Methodology**	**Sample Size**	**Response Rate **^d^	**Additional Findings**

Smith et al [28]	2004	China	16%	32%	0%	Hand Delivered	286	79%	Physicians younger than 25 had the lowest smoking rate
Soto Mas et al [29]	2003	United States ^e^	7%	-	-	Postal Survey	45	56%	No physicians reported being current *cigarette *smokers
Kenna & Wood [30]	2002	United States	4%	-	-	Postal Survey	104	63%	Fewer physicians smoked when compared to dentists
Pärna et al [31,32]	2002	Estonia	-	25%	11%	Postal Survey	2668	68%	Twice as many males as females were ex-smokers
Hodgetts et al [33]	2002	Bosnia & Herzegovina	40%	-	-	Hand Delivered	112	73%	Fewer physicians smoked when compared to nurses
Gunes et al [34]	2002	Turkey	38%	-	-	Hand Delivered	257	85%	Around one-fifth of smokers were only occasional smokers
Nollen et al [35]	2002	Nigeria	3%	-	-	Hand Delivered	373	60%	Smoking rates in two different hospitals were the same
Misra & Vadaparampil [36]	2002	United States ^f^	3%	-	-	Postal Survey	254	37%	The smoking status of a further 6% of physicians was not defined
Barengo et al [37]	2001	Finland	-	5%	3%	Postal Survey	3057	69%	Occasional smoking was more common among male physicians
Kannegaard et al [38]	2001	Denmark	15%	-	-	Postal Survey	729	75%	The physicians' smoking rate fell 4% between 1999 and 2001
Ahmadi et al [39]	2001*	Iran	9%	-	-	Hand Delivered	111	n/s^g^	Residents had a higher smoking rate than attending physicians
Pizzo et al [40]	2000	Italy	28%	32%	20%	Telephone Survey	526	72%	Physician smoking rates differed by geographical region
Ohida et al [41]	2000	Japan	-	27%	7%	Postal Survey	3771	84%	Male physicians aged 40–49 had the highest smoking rate
An et al [42]	2000	United States	2%	-	-	Postal Survey	750	61%	A further 17% of physicians had ever smoked in the past
John & Hanke [43]	1999	Germany	18%	-	-	Census Data	1144	79%	Fewer physicians smoked when compared to nurses
La Vecchia et al [44]	1999	Italy	24%	25%	23%	Interview	501	n/s	Physicians aged 41–50 had the highest smoking rate
Power et al [45]	1999	Ireland	16%	-	-	Telephone Survey	171	85%	Most physicians understood the dangers of smoking
Williang et al [46]	1999	Denmark	25%	-	-	Postal Survey	445	91%	Fewer physicians smoked when compared to nurses
McEwan & West [47]	1999	United Kingdom	4%	-	-	Postal and Telephone	303	75%	Most physicians felt they should advise patients to quit
Nardini et al [48]	1998*	Italy	39%	-	-	Hand Delivered	959	57%	Fewer physicians smoked when compared to nurses
Josseran et al [49]	1998	France	32%	34%	25%	Telephone Survey	2073	67%	Physicians older than 40 had the highest smoking rate
Hepburn et al [50]	1997	United States	11%	-	-	Postal Survey	150	65%	More than half of the smokers used smokeless tobacco
Kawahara et al [51]	1997	Japan	26%	28%	5%	Postal Survey	709	91%	Physicians aged 40–49 years had the highest smoking rate
Samuels [52]	1996	Israel	16%	16%	15%	Interview	260	87%	The highest smoking rate was seen among radiologists
Zanetti et al [53]	1996	Italy	31%	29%	34%	Hand Delivered	2453	68%	Fewer physicians smoked when compared to nurses
Hay [54]	1996	New Zealand	5%	5%	5%	Census Data	7335	97%^h^	Fewer physicians smoked when compared to nurses
Li et al [55]	1996	China	45%	61%	12%	Hand Delivered	493	82%	Smoking rates have increased dramatically in recent years
Young & Ward [56]	1996	Australia	3%	4%	2%	Postal Survey	855	67%	Older physicians were more likely to be current smokers
Roche et al [57]	1996*	Australia	4%	-	-	Postal Survey	908	55%	A further 8% said they had previously smoked tobacco
Roche et al [58]	1995*	Australia	6%	6%	5%	Postal Survey	1365	55%	Trainee psychiatrists were more likely to be smokers
Barengo et al [59]	1995	Finland	-	7%	3%	Postal Survey	1221	76%	Male physicians older than 45 had the highest smoking rate
Nardini et al [60]	1995	Italy	25%	-	-	Conference Survey	605	62%	Physicians aged 40–50 years had the highest smoking rate
Hill & Braithwaite [61]	1994	United States ^i^	4%	-	-	Postal Survey	121	32%	Fewer physicians smoked when compared to dentists
Kawane & Soejima [62]	1994	Japan	29%	-	-	Hand Delivered	163	60%	Younger physicians had the highest smoking rates
Josseran et al [63]	1994	France	34%	36%	25%	Telephone Survey	1013	65%	Male physicians were also heavier smokers than female physicians
Kawakami et al [64]	1994	Japan	21%	24%	7%	Postal Survey	323	71%	Only 60% of smokers intended to reduce or quit their habit in future
Grossman et al [65]	1994	Costa Rica	19%	-	-	Hand Delivered	217	76%	88% of smokers intended to reduce or quit their habit in future
Frank et al [66–68]	1994	United States	-	-	4%	Postal Survey	4501	59%	Fewer family physicians smoked than physicians, generally
Tapia-Conyer et al [69]	1993	Mexico	27%	30%	21%	Postal Survey	3488	98%	Physicians aged 33–43 years had the highest smoking rate
Polyzos et al [70]	1992	Greece	49%	-	-	Hand Delivered	148	n/s	Surgeons had a higher smoking rate than internists
Heloma et al [71]	1992	Finland	10%	-	-	Postal Survey	725	72%	More physicians smoked when compared to nurses
De Koninck et al [72]	1992	Canada	-	13%	7%	Postal Survey	1540	51%	Over half of all male physicians had previously smoked
Bener et al [73]	1992	Arab Emirates	36%	44%	8%	Postal Survey	275	92%	Almost half the smokers were aged over 45 years
Tessier et al [74]	1991	France	21%	22%	14%	Postal Survey	4318	37%	Over half had made at least one attempt to quit smoking
Hussain et al [75]	1991	United Kingdom	5%	-	-	Postal Survey	1069	82%	Fewer physicians smoked when compared to nurses
Yaacob & Abdullah [76]	1991	Malaysia	18%	25%	0%	Postal and Hand Delivered	120	100%	Around half the smokers had begun before medical school
Doll et al [77]	1990	United Kingdom	-	18%	-	Postal Survey	10807	94%	A large proportion of smokers only smoked pipes and cigars
Kaetsu et al [78]	1990	Japan	32%	33%	5%	Postal Survey	3565	63%	Male physicians younger than 40 had the highest smoking rate
Jormanainen et al [79]	1990	Finland	-	10%	6%	Postal Survey	1231	76%	General practitioners had a higher smoking rate than specialists
Brink et al [80]	1990	United States	2%	-	-	Postal Survey	132	77%	Physicians smoked at similar rates when compared to dentists
Bener et al [73]	1990	Kuwait	38%	45%	16%	Postal Survey	252	84%	Over half the smokers were aged 35 to 44 years
Hensrud & Sprafka [81]	1990	United States	9%	10%	2%	Postal Survey	393	83%	Physicians aged 60–69 years had the highest smoking rate
Waalkens et al [82]	1989	The Netherlands	32%	37%	14%	Postal Survey	362	63%	More consultants smoked than house officers
Kawane [83–85]	1989	Japan	25%	26%	6%	Postal Survey	3640	59%	Chest physicians smoked at lower rates than physicians, generally
Dekker et al [86]	1989	Netherlands	38%	41%	24%	Postal Survey	263	82%	More general practitioners smoked than consultants
Hughes et al [87]	1989	United States	6%	-	-	Postal Survey	5426	59%	Older physicians were more likely to have ever smoked
Scott et al [88]	1988	United States	5%	5%	4%	Postal Survey	2341	86%	Physicians aged 55–64 years had the highest smoking rate
Fowler et al [89]	1988	United Kingdom	4%	-	-	Postal Survey	2176	75%	A further 11% of male physicians smoked pipes or cigars
Saeed [90]	1987	Saudi Arabia	34%	-	-	Hand Delivered	716	81%	Males smoked more sticks per day than female physicians
Nutbeam & Catford [91]	1987	Wales	14%	17%	0%	Postal Survey	310	60%	Almost one-quarter of female physicians were ex-smokers
Hughes et al [92]	1987	United States	4%	-	-	Postal Survey	1754	60%	Psychiatry residents had the highest smoking rate
Davies & Rajan [93]	1987	United Kingdom	3%	-	-	Postal Survey	94	72%	Fewer physicians smoked when compared to nurses
Cheng & Lam [94]	1987	Hong Kong	5%	7%	0%	Postal Survey	133	88%	Only 8% of female physicians had ever smoked tobacco
Stillman et al [95]	1987	United States	6%	-	-	Postal Survey	6050	69%	A no-smoking hospital policy helped reduce the smoking rate
Sarkar et al [96]	1987	India	32%	48%	3%	Interview	218	99%	Physicians aged 20–29 had the highest smoking rate
Franceschi et al [97]	1985	Italy	31%	-	-	Postal and Telephone	709	86%	Over half of the smokers reported no attempt to quit smoking
Linn et al [98]	1984	United States	4%	-	-	Postal and Telephone	211	67%	A further 2% smoked either weekly or monthly
Joossens et al [99]	1983	Belgium	32%	34%	16%	Postal Survey	2157	67%	Around half of the smokers were evaluated as being dissonant
Kaetsu et al [100]	1983	Japan	43%	45%	9%	Postal Survey	4232	84%	Male physicians younger than 40 had the highest smoking rate
Sachs [101,102]	1983	United States	12%	-	-	Conference Survey	594	27%	Smoking was higher among non-practicing specialists
Seiler [103]	1983*	Scotland	19%	-	-	Postal Survey	607	81%	Almost half of smoking doctors had spouses who also smoked
Senior [104]	1982*	Canada	19%	-	-	Hand Delivered	88	52%	Fewer physicians smoked when compared to nurses
Fortmann et al [105]	1982	United States	8%	-	-	Postal Survey	221	62%	Physicians older than 46 years had the highest smoking rate
Hay [106]	1981	New Zealand	15%	15%	13%	Census Data	4937	97%^h^	Fewer physicians smoked when compared to nurses
Ballal [107]	1980	Sudan	-	46%	1%	Postal and Hand Delivered	753	72%	Some respiratory symptoms were more common among smokers
Wyshak et al [108]	1979	United States	14%	-	-	Postal Survey	289	70%	Fewer physicians smoked when compared to lawyers
Wells et al [109]	1978	United States	-	15%	-	Hand Delivered	151	76%	Surgeons/obstetricians had the highest smoking rates
Dodds et al [110]	1977	Australia	21%	22%	16%	Postal and Telephone	275	80%	Physicians aged 50–59 years had the highest smoking rate
Hay [111]	1976	New Zealand	-	20%	17%	Census Data	4089	97%^h^	Obstetricians had the highest smoking rates of all
Aarø et al [112]	1974	Norway	-	35%	22%	Postal Survey	1138	95%	Male physicians aged 55–64 had the highest smoking rate
Rankin et al [113]	1974	Australia	14%	14%	17%	Postal Survey	1276	69%	Physicians aged 50–59 years had the highest smoking rate

^a ^Including the reference number as listed in this manuscript, ^b ^Year in which the study was undertaken – not the year of publication (Studies that continued over more than one year list the latest year. In cases where the study year was not listed, manuscripts are arranged by publication year and marked with an asterisk*), ^c ^Smoking rates rounded to the nearest whole number, ^d ^Response rates rounded to the nearest whole number (as some studies investigated multiple occupational groups, response rates may be indicative of the entire group rather than just physicians), ^e ^Subjects were restricted to Hispanic physicians living in the United States, ^f ^Subjects were restricted to Asian-Indian physicians living in the United States, ^g ^The survey used a convenience sample with an unspecified response rate, ^h ^Response rate of the entire census, ^i ^Subjects were restricted to African-American physicians living in the United States

One confounding factor across many investigations however, was a lack of standardisation regarding the definition of 'current smoker'. Although most authors referred to their subjects as simply being either smokers or non-smokers, some used recall periods ranging from one week to one month in their definition of the term 'current'. Others listed no recall period. This may have arisen due to the inherent difficulties in assessing smoking habits over time, and the fact that most investigations simply described the point-prevalence of tobacco smoking among the surveyed group. Not all physicians smoked cigarettes either, with a study of Hispanic physicians in the United States [29] finding that 7% of their subjects smoked cigars, and none smoked cigarettes. In 1990 Doll et al [77] also revealed that a large proportion of their British physicians only smoked pipes or cigars, similar to Fowler et al's [89] earlier finding in the same country. Another confounding factor was that some studies appeared to use convenience samples, rather than true random sampling. Furthermore, a certain proportion of manuscripts did not clearly describe their sample group or their entire research methodology in detail. Nevertheless, such investigations were in the minority, with a large proportion of all manuscripts located during this review having reasonable sample sizes in the hundreds, and sufficiently high response rates to allow confidence in the published data.

## Discussion

A large proportion of all research on physicians' tobacco smoking (52 of 81 studies) appears to have been conducted since 1990 [28-81]. Twenty-three studies had been undertaken between 1980 and 1989 [82-107], with the remaining 6 investigations conducted prior to 1980 [108-113]. By country, 18 manuscripts in this review originated from the United States, 7 from Japan, 6 from Italy, 5 from the United Kingdom, 5 from Australia, 3 from New Zealand, and the remainder from other areas. When investigated from an international perspective, the overall prevalence of physician's smoking appears to have followed two distinct trends during this time. First of all, most developed countries appear to have experienced a steady decline in physicians' smoking rates over the past 30 years. Since the year 2000 for example, four separate studies [29,30,36,42] have shown the prevalence of smoking among American physicians to be lower than 10%.

Three investigations of their Australian counterparts in the 1990s [56-58] revealed a prevalence of around 5%, while in New Zealand, analysis of census data also suggested a similar rate during this period [54]. Tobacco use among British physicians has been well-studied longitudinally [10,11,77], although their smoking rate does not appear to be as low as the abovementioned countries, possibly due to the relatively large number who continue to smoke pipes and cigars, rather than cigarettes. Nevertheless, overall tobacco consumption has still declined markedly, with Doll et al [77] revealing that the absolute proportion of British physicians who smoked cigars, pipes or cigarettes fell from 62% to 18% between 1951 and 1990. As the British Doctor's Study follows the same cohort longitudinally, this represents one of the clearest reductions in absolute tobacco smoking rates among physicians.

According to our review, such trends may not be uniform across all countries however, with physicians in some developed regions still smoking at fairly high rates. Multiple investigations from Italy [40,48,53], Japan [41,51,62,78,83-85,100] and France [49,63] for example, have consistently documented smoking prevalence rates over 25%. A second trend is also evident in some newly developing countries, where contemporary physicians appear to be smoking at high rates in China [55], Estonia [31,32], Bosnia/Herzegovina [33] and Turkey [34]. In China for example, Li et al [55] reported that tobacco smoking rates among physicians have actually been increasing in recent years. A surprisingly low rate was found in Nigeria however (3%) [35], suggesting that exceptions are still possible in this latter group. The lowest overall smoking rate was documented in the United States (2%) [42,80], with similar low rates also being demonstrated in Australia (3%) [56] and the United Kingdom (3%) [93]. The highest smoking prevalence rate was recorded in Greece [70], where roughly half of all physicians (49%) reported themselves to be current smokers. Almost half of all Chinese (45%) [55] and Japanese physicians (43%) [100] were revealed to be current smokers in two separate studies. Similar results were also documented in Kuwait (38%) and the United Arab Emirates (36%) [73], particularly among males (among whom 45% and 44% smoked, respectively). Almost half (48%) of all male Indian physicians from one study [96] were smoking. The proportion of ex-smoking physicians is also worth considering, with prevalence rates of 8% in Australia [57], 17% in the United States [42], 23% in Wales [91] and 52% in Canada [72], being previously documented.

For current smokers by gender, the highest smoking prevalence rates were 61% among male physicians in China [55] and 34% among female physicians in Italy [53]. Two investigations from France also found that one-quarter of their female physicians smoked tobacco on a regular basis [49,63]. Conversely, other research from China [28], Malaysia [76], Wales [91] and Hong Kong [94] revealed no female smokers at all. This may suggest a cultural reluctance for professional women to smoke in certain regions, such as Asia. An important observation during our review was the relatively large number of studies where male physicians smoked at higher rates than their female counterparts. This finding was not without exception however. In Italy for example, Zanetti et al [53] found that more women doctors smoked than men, while in Israel [52], Australia [58] and the United States [88], smoking prevalence rates were almost the same between the genders. A large proportion of manuscripts did not divide their smoking prevalence rates by sex however, making it impossible to do further gender comparisons. Many authors documented age-related differences in physicians' smoking rates, with older physicians for the most part, more likely to be current smokers. Nevertheless, in China [28], Japan [62,100], Mexico [69] and India [96], tobacco usage was actually more prevalent in younger physicians. This latter result suggests that a challenge for public health policy makers in countries with high smoking rates among young physicians, still lies ahead.

Aside from overall prevalence rates, additional useful information was also obtained with regard to physician's tobacco smoking habits. Firstly, some studies simultaneously investigated the tobacco usage habits of dentists, nurses and other hospital staff while surveying doctors. Two investigations from the United States [30,61] found that fewer physicians smoked when compared to dentists, while another study demonstrated very similar, albeit very low, smoking rates among the two professional groups [80]. In 1979, Wyshak et al [108] found that physicians were less likely to be current smokers than lawyers. Most studies found that fewer physicians smoked when compared to nurses at the same facility, although an investigation from Finland [71] suggested the opposite situation may sometimes occur. Even so, physicians in most societies tend to give up smoking before other occupational groups and the general public for a number of reasons [15]. Firstly, they may recognise the negative medical consequences more quickly than the general public. Secondly, their devotion to health naturally conflicts with unhealthy behaviours. Thirdly, tobacco smoking usually incurs a negative image in the health-care profession long before it does so in the wider community [15]. In this regard, doctors are well-equipped to evaluate scientific knowledge, and can be reasonably expected to act upon new discoveries, if warranted [114]. Furthermore, smoking rates in developed countries tend to decrease over time due to a generational effect, as the social climate of a country changes and more people give up smoking, doctors included [86].

Tobacco smoking by medical speciality also revealed some interesting, though inconsistent, results during this review. One study for example, found that family physicians smoked less than physicians generally [67], while two others suggested that general practitioners smoked more often than specialists [79,86]. In the Netherlands, more consultants smoked than house officers [82]. Trainee psychiatrists [58] and psychiatry residents [92] were the most likely to smoke in some investigations, while in others it was surgeons [70], obstetricians [111] or surgeons and obstetricians [109]. Encouragingly, Kawane et al [83-85] demonstrated that Japanese chest physicians had a lower smoking rate than Japanese physicians, generally. Exactly how much a physician's medical speciality influences their smoking habits is uncertain. A previous study of Malaysian doctors for example [76] found that around half were already smoking before they entered medical school. Based on the findings of multiple investigations therefore, it is very difficult to ascertain which medical speciality actually has the highest or lowest smoking rate.

Regarding antismoking practices, most physicians in a British study [47] felt they should advise patients to quit, and in France [74] over half the tobacco using physicians had made at least one serious attempt to quit smoking themselves. In Italy however [97], more than half the physicians who currently smoked had made no attempt to quit smoking, and in Japan [64] only 60% of smokers stated any intention of reducing their tobacco consumption or quitting altogether. Other authors have already suggested that Japanese physicians may not be setting a good example in this regard [115]. The institutions where doctors work may also play an important role in tobacco control, with an American study [95] demonstrating that a hospital no-smoking policy was useful in helping to reduce the overall smoking rate among staff. Hospitals in the United States were the first industry to declare a national smoking ban in the early 1990s, and ones which later influenced social norms and probably reduced overall smoking rates [116]. Even so, the actual hospital in which physicians work, as well as the geographical location where they live, may not always affect the smoking rates of physicians in the same country. One Italian study for example, found different smoking rates by region [40], although in Nigeria [35] smoking rates of physicians in two different hospitals were exactly same, with both being encouragingly low (3%). A doctor's smoking habit may also be associated with the smoking habits of their spouse. Previous studies from Scotland [103] and New Zealand [117] for example, revealed that around half of all male physicians who smoked, also had smoking wives. This situation may reflect assortative mating and its subsequent influence on partner choice, at least with regard to smoking.

How much a physician's personal smoking behaviour affects their professional attitude and clinical behaviour represents a critical issue in public health policy, as physicians are on the frontlines of primary health care. Although medical professionals have many opportunities to reduce the prevalence of smoking among their patients, physicians may have not yet maximized their efforts in meeting the tobacco epidemic. Doctors incur a certain responsibility as exemplars for patients with regard to healthy behaviour [118], as well as the public image they inadvertently portray outside of the work environment [119]. Having any physicians who smoke may increase public scepticism, with people inclined to ask why should they stop smoking when their doctor continues to do so? [120]. Continued tobacco usage by health care workers undermines the message to smokers that quitting is important [121], and as early as 1976 it was suggested that physicians could best persuade patients to quit if they themselves did not smoke [5]. In 1983, Sachs [101] stated that 80% of US citizens expected their physicians to be non-smokers, and in 1984, Wells et al [109] suggested that physicians with good personal health habits counselled their patients significantly more about all health habits. As physicians gain more insight into their own health and health habits, their advice to patients becomes increasingly relevant and effective [122]. Although methods for treating tobacco dependence in clinical practice have been described elsewhere [123-125], the entire process need not be overly taxing for physicians. At the most basic level, such interventions may require them to ask only two questions: 'do you smoke?' and 'do you want to quit?' [123]. Nevertheless, such guidelines are not always followed for a variety of reasons.

The extent to which the professional practice of physicians is affected by their own smoking habits is very important for policymakers, and has been examined in certain investigations. One of the most marked differences in this regard was found in Greece [70], where only half the smoking physicians were involved in smoking cessation counselling, compared to 100% of their non-smoking colleagues. Several Japanese studies revealed differences in smoking-cessation advice [64] and taking a patients' smoking history [51], with both being significantly more commonplace among non-smoking physicians. Similar findings were also seen in Finland [37], while Pärna et al [31] revealed that Estonian physicians who smoked were reluctant to disturb a patient's privacy by asking about their tobacco usage. Knowledge of smoking-related damage also showed correlations with smoking behaviour in an Italian study [48], although their analysis included other health professionals as well as physicians.

Not all studies revealed differences however. In Israel for example, Samuels [52] asked physicians whether or not they advised patients to stop smoking during consultations, and found no difference between smokers and non-smokers. A longitudinal study of Chinese physicians also revealed that the effects of smoking on counselling behaviour varied between 1987 and 1996 [55]. In 1987 for example, smoking behaviour was an influential factor, whereas by 1996 it had ceased to be so. Other confounding issues were raised by the Chinese study. Firstly, only one-third of physicians believed that they were the most influential person who could help patients quit. On the other hand, over three-quarters of them believed that physicians can set a good example for patients by not smoking. Most disturbingly, anti-smoking counselling practices appear to be diminishing among Chinese physicians in recent years, while their overall prevalence of smoking is probably increasing during the same time [55]. These discrepancies between various countries suggest that not only are physician-targeted smoking interventions urgently needed in public health, but that they should also be culturally specific.

Although our current review sourced a wide variety of manuscripts from many countries, there are a few limitations worth considering. Firstly, for practical reasons, only English language papers were included. With such a methodology, it is possible that we may have missed some manuscripts published in domestic journals and local languages. The decision to restrict our study to English language papers was a purely pragmatic one, however. We felt that formulating inclusion criteria for other languages would have been too difficult to practically achieve, given the wide variety of dialects in which contemporary research is now being published. Furthermore, including some languages but not others would also have led to a bias against manuscripts written in languages that the authors could not understand. Secondly, we assumed that, knowing the topic's importance, significant domestic findings would most likely have been published in an international, English language journal.

Nevertheless, the nature of biomedical publication itself is known to incur some inherent bias against developing countries, and this will probably be reflected in the overall number of publications available from those regions. As previously noted by Rahman and Fukui [126], the imbalance between developing and developed countries in terms of biomedical publication is not only significant, but the total share of publications originating from low-income countries may also be declining in recent years. This limitation in data equity makes it difficult to unequivocally decide why smoking rates differ between physicians in developed and developing countries. Given that such limitations occur, it is important that continued measures be taken to encourage local researchers, irrespective of nationality, to publish their findings in a globally accessible format. Furthermore, it is imperative that the knowledge gained from international smoking research be equally and consistently disseminated to physicians and other public health policy makers in all countries.

A further difficulty encountered during this review was ascertaining exactly how comparable the smoking prevalence data is from country to country, and also from study to study in the same country. As previously mentioned, one issue we encountered early on was the definition of the term 'smoker' that researchers had actually used. Another issue is reflected in the changing nature of survey methodologies, even in the same country, combined with the fact that many surveys are not originally designed for a comparative analysis of smoking rates at a later date; a point acknowledged by certain authors of longitudinal data [43]. Probably the best study with longitudinal smoking data is still the British Doctor's Study [77], which has followed the same cohort of physicians for over 50 years. Future researchers investigating the smoking habits of health care workers would do well to follow this pioneering model.

## Conclusion

Regardless of what country the data originates from, tobacco smoking by physicians remains a contentious issue in public health. Global policy making demands accurate data on this topic, from which at least two distinct benefits can be derived. Firstly, this kind of data can help predict how effective any potential anti-smoking campaigns may actually be in a particular country [15]. That is, it would be difficult to convince the general public not to smoke if their physician role-models continue to do so. Secondly, and perhaps most importantly, it allows public health policy makers to determine how 'mature' a country's smoking epidemic currently is, and thus, how soon the overall community prevalence rate could be expected to decline. The current paper presented an international review of all modern literature concerning tobacco usage patterns within the medical profession. Overall, our review suggests that smoking rates in this professional group vary widely from region to region. International policy makers who are attempting to tackle the tobacco problem on a global scale, such as the WHO, will need to carefully consider these regional differences when devising potential control strategies. In this regard, high smoking rates among doctors in some countries for example, suggests that physicians may not always be the best role models from where sound policy can actually originate.

On the other hand, comparison of physician's smoking rates with other health professionals suggests that fewer doctors seem to be smoking when compared to nurses in the same countries. In addition to physicians, it has also been shown that smoking is now becoming quite rare among dentists in many areas [127]. Dentists may therefore be ideally placed to work with physicians in helping to reduce the overall burden of tobacco use among patients. A multidisciplinary effort from all health care workers would seem to be an ideal goal, from a global perspective. Nevertheless, the fact that any health care workers smoke at all is unfortunate, given their undoubted status as public health exemplars. As such, further preventive efforts will need to be focussed on the personal health behaviours of physicians, particularly those in developing countries. To assist in the promotion of sound public health policy, it is important that physician's tobacco usage continues its decline in future years, so that the medical profession can remain at the forefront of anti-smoking programs and lead the way as public health exemplars in the 21^st ^century.

## Competing interests

The author(s) declare that they have no competing interests.

## Authors' contributions

DRS conceived the idea for the paper. DRS and PAL contributed equally to the literature search. Both DRS and PAL contributed to the evaluation of the literature. DRS drafted the manuscript. Both authors reviewed and approved the final manuscript.

## Pre-publication history

The pre-publication history for this paper can be accessed here:



## References

[B1] World Health Organization (WHO) Why is tobacco a public health priority?.

[B2] Centers for Disease Control and Prevention (CDC) (1993). Physician and other health-care professional counseling of smokers to quit – United States, 1991. MMWR Morb Mortal Wkly Rep.

[B3] Fowler G (1993). Educating doctors in smoking cessation. Tob Control.

[B4] Nett LM (1990). The physician's role in smoking cessation: A present and future agenda. Chest.

[B5] Garfinkel L (1976). Cigarette smoking among physicians and other health professionals, 1959–1972. CA Cancer J Clin.

[B6] Håkansta C (2004). Workplace Smoking Working Paper: A review of national and local practical and regulatory measures.

[B7] Doll R, Hill AB (1954). The mortality of doctors in relation to their smoking habits: A preliminary report. BMJ.

[B8] Doll R, Hill AB (2004). The mortality of doctors in relation to their smoking habits: A preliminary report. 1954. BMJ.

[B9] Stampfer M (2004). New insights from the British doctors study. BMJ.

[B10] Doll R, Peto R, Boreham J, Sutherland I (2004). Mortality in relation to smoking: 50 years' observations on male British doctors. BMJ.

[B11] Doll R, Peto R, Boreham J, Sutherland I (2005). Mortality from cancer in relation to smoking: 50 years observations on British doctors. Br J Cancer.

[B12] Hammond EC, Horn D (1958). Smoking and death rates – Report on forty-four months of follow-up of 187,783 men. I: Total mortality. JAMA.

[B13] Hammond EC, Horn D (1958). Smoking and death rates – Report on forty-four months of follow-up of 187,783 men. II: Death rates by cause. JAMA.

[B14] Hammond EC, Horn D (1984). Landmark article March 15, 1958: Smoking and death rates – Report on forty-four months of follow-up of 187,783 men. By E. Cuyler Hammond and Daniel Horn. JAMA.

[B15] Davis RM (1993). When doctors smoke. Tob Control.

[B16] Gardner MN, Brandt AM (2006). The Doctors' Choice Is America's Choice: The Physician in US Cigarette Advertisements, 1930–1953. Am J Public Health.

[B17] Kawane H (1993). When doctors advertised cigarettes. Tob Control.

[B18] Centers for Disease Control and Prevention (CDC) (1977). Smoking behavior and attitudes of physicians, dentists, nurses, and pharmacists, 1975. MMWR Morb Mortal Wkly Rep.

[B19] Sterling TD, Weinkam JJ (1976). Smoking characteristics by type of employment. J Occup Med.

[B20] Garfinkel L, Stellman SD (1986). Cigarette smoking among physicians, dentists, and nurses. CA Cancer J Clin.

[B21] Stellman SD, Boffetta P, Garfinkel L (1988). Smoking habits of 800 000 American men and women in relation to their occupations. Am J Ind Med.

[B22] Nelson DE, Giovino GA, Emont SL, Brackbill R, Cameron LL, Peddicord J (1994). Trends in cigarette smoking among US physicians and nurses. JAMA.

[B23] Nelson DE, Emont SL, Brackbill RM, Cameron LL, Peddicord J, Fiore MC (1994). Cigarette smoking prevalence by occupation in the United States. A comparison between 1978 to 1980 and 1987 to 1990. J Occup Med.

[B24] Lee DJ, LeBlanc W, Fleming LE, Gomez-Marin O, Pitman T (2004). Trends in US smoking rates in occupational groups: The National Health Interview Survey 1987–1994. J Occup Environ Med.

[B25] van Reek J, Adriaanse H (1991). Smoking by physicians in Scandinavia: 1952–1989. Scand J Soc Med.

[B26] Adriaanse H, van Reek J, Metsemakers J (1986). Smoking behaviour of Dutch general practitioners in the period 1977–1983. Scand J Prim Health Care.

[B27] Adriaanse H, Halfens R, Drop MJ, van Reek J (1985). Physicians, smoking, and health in the Netherlands. NY State J Med.

[B28] Smith DR, Wei N, Zhang YJ, Wang RS (2006). Tobacco smoking habits among a cross-section of rural physicians in China. Aust J Rural Health.

[B29] Soto Mas FG, Papenfuss RL, Jacobson HE, Hsu CE, Urrutia-Rojas X, Kane WM (2005). Hispanic physicians' tobacco intervention practices: A cross-sectional survey study. BMC Public Health.

[B30] Kenna GA, Wood MD (2005). The prevalence of alcohol, cigarette and illicit drug use and problems among dentists. J Am Dent Assoc.

[B31] Parna K, Rahu K, Rahu M (2005). Smoking habits and attitudes towards smoking among Estonian physicians. Public Health.

[B32] Parna K, Rahu K, Barengo NC, Rahu M, Sandstrom PH, Jormanainen VJ (2005). Comparison of knowledge, attitudes and behaviour regarding smoking among Estonian and Finnish physicians. Soz Praventivmed.

[B33] Hodgetts G, Broers T, Godwin M (2004). Smoking behaviour, knowledge and attitudes among Family Medicine physicians and nurses in Bosnia and Herzegovina. BMC Fam Pract.

[B34] Gunes G, Karaoglu L, Genc MF, Pehlivan E, Egri M (2005). University hospital physicians' attitudes and practices for smoking cessation counseling in Malatya, Turkey. Patient Educ Couns.

[B35] Nollen NL, Adewale S, Okuyemi KS, Ahluwalia JS, Parakoyi A (2004). Workplace tobacco policies and smoking cessation practices of physicians. J Natl Med Assoc.

[B36] Misra R, Vadaparampil ST (2004). Personal cancer prevention and screening practices among Asian Indian physicians in the United States. Cancer Detect Prev.

[B37] Barengo NC, Sandstrom HP, Jormanainen VJ, Myllykangas M (2005). Attitudes and behaviours in smoking cessation among general practitioners in Finland 2001. Soz Praventivmed.

[B38] Kannegaard PN, Kreiner S, Gregersen P, Goldstein H (2005). Smoking habits and attitudes to smoking 2001 among hospital staff at a Danish hospital – Comparison with a similar study 1999. Prev Med.

[B39] Ahmadi J, Khalili H, Jooybar R, Namazi N, Aghaei PM (2001). Cigarette smoking among Iranian medical students, resident physicians and attending physicians. Eur J Med Res.

[B40] Pizzo AM, Chellini E, Grazzini G, Cardone A, Badellino F (2003). Italian general practitioners and smoking cessation strategies. Tumori.

[B41] Ohida T, Sakurai H, Mochizuki Y, Kamal AM, Takemura S, Minowa M (2001). Smoking prevalence and attitudes toward smoking among Japanese physicians. JAMA.

[B42] An LC, Bernhardt TS, Bluhm J, Bland P, Center B, Ahluwalia JS (2004). Treatment of tobacco use as a chronic medical condition: Primary care physicians' self-reported practice patterns. Prev Med.

[B43] John U, Hanke M (2003). Tobacco-smoking prevalence among physicians and nurses in countries with different tobacco-control activities. Eur J Cancer Prev.

[B44] La Vecchia C, Scarpino V, Malvezzi I, Baldi G (2000). A survey of smoking among Italian doctors. J Epidemiol Community Health.

[B45] Power B, Neilson S, Perry IJ (2004). Perception of the risks of smoking in the general population and among general practitioners in Ireland. Ir J Med Sci.

[B46] Willaing I, Jørgensen T, Iversen L (2003). How does individual smoking behaviour among hospital staff influence their knowledge of the health consequences of smoking?. Scand J Public Health.

[B47] McEwen A, West R (2001). Smoking cessation activities by general practitioners and practice nurses. Tob Control.

[B48] Nardini S, Bertoletti R, Rastelli V, Ravelli L, Donner CF (1998). Personal smoking habit and attitude toward smoking among the health staff of a general hospital. Monaldi Arch Chest Dis.

[B49] Josseran L, King G, Guilbert P, Davis J, Brucker G (2005). Smoking by French general practitioners: Behaviour, attitudes and practice. Eur J Public Health.

[B50] Hepburn MJ, Johnson JM, Ward JA, Longfield JN (2000). A survey of smoking cessation knowledge, training, and practice among U.S. Army general medical officers. Am J Prev Med.

[B51] Kawahara K, Ohida T, Osaki Y, Mochizuki Y, Minowa M, Yamaguchi N (2000). Study of the smoking behavior of medical doctors in Fukui, Japan and their antismoking measures. J Epidemiol.

[B52] Samuels N (1997). Smoking among hospital doctors in Israel and their attitudes regarding anti-smoking legislation. Public Health.

[B53] Zanetti F, Gambi A, Bergamaschi A, Gentilini F, De Luca G, Monti C (1998). Smoking habits, exposure to passive smoking and attitudes to a non-smoking policy among hospital staff. Public Health.

[B54] Hay DR (1998). Cigarette smoking by New Zealand doctors and nurses: Results from the 1996 population census. NZ Med J.

[B55] Li HZ, Fish D, Zhou X (1999). Increase in cigarette smoking and decline of anti-smoking counselling among Chinese physicians: 1987–1996. Health Prom Int.

[B56] Young JM, Ward JE (1997). Declining rates of smoking among medical practitioners. Med J Aust.

[B57] Roche AM, Parle MD, Saunders JB (1996). Managing alcohol and drug problems in general practice: A survey of trainees' knowledge, attitudes and educational requirements. Aust NZ J Public Health.

[B58] Roche AM, Parle MD, Stubbs JM, Hall W, Saunders JB (1995). Management and treatment efficacy of drug and alcohol problems: What do doctors believe?. Addiction.

[B59] Barengo NC, Sandstrom PH, Jormanainen VJ, Myllykangas MT (2004). Changes in smoking prevalence among Finnish physicians 1990–2001. Eur J Public Health.

[B60] Nardini S, Bertoletti R, Rastelli V, Donner CF (1998). The influence of personal tobacco smoking on the clinical practice of Italian chest physicians. Eur Respir J.

[B61] Hill HA, Braithwaite RL (1997). Attitudes, beliefs, and practices regarding smoking and smoking cessation among African-American physicians and dentists. J Natl Med Assoc.

[B62] Kawane H, Soejima R (1996). Smoking among doctors in a medical school hospital. Kawasaki Med J.

[B63] Josseran L, King G, Velter A, Dressen C, Grizeau D (2000). Smoking behavior and opinions of French general practitioners. J Natl Med Assoc.

[B64] Kawakami M, Nakamura S, Fumimoto H, Takizawa J, Baba M (1997). Relation between smoking status of physicians and their enthusiasm to offer smoking cessation advice. Intern Med.

[B65] Grossman DW, Knox JJ, Nash C, Jimenez JG (1999). Smoking: Attitudes of Costa Rican physicians and opportunities for intervention. Bull World Health Organ.

[B66] Frank E (1995). The Women Physicians' Health Study: Background, objectives, and methods. J Am Med Womens Assoc.

[B67] Frank E, Lutz LJ (1999). Characteristics of women US family physicians. Arch Fam Med.

[B68] Frank E, Rothenberg R, Lewis C, Belodoff BF (2000). Correlates of physicians' prevention-related practices. Findings from the Women Physicians' Health Study. Arch Fam Med.

[B69] Tapia-Conyer R, Cravioto P, de la Rosa B, Galvan F, Garcia-de la Torre G, Kuri P (1997). Cigarette smoking; knowledge and attitudes among Mexican physicians. Salud Publica Mex.

[B70] Polyzos A, Gennatas C, Veslemes M, Daskalopoulou E, Stamatiadis D, Katsilambros N (1995). The smoking-cessation promotion practices of physician smokers in Greece. J Cancer Educ.

[B71] Heloma A, Reijula K, Tikkanen J, Nykyri E (1998). The attitudes of occupational health personnel to smoking at work. Am J Ind Med.

[B72] De Koninck M, Guay H, Bourbonnais R, Bergeron P (1995). Physical, mental, and reproductive health of Quebec women physicians. J Am Med Womens Assoc.

[B73] Bener A, Gomes J, Anderson JAD (1993). Smoking habits among physicians in two gulf countries. J R Soc Health.

[B74] Tessier JF, René L, Nejjari C, Belougne D, Moulin J, Freour P (1993). Attitudes and opinions of French general practitioners towards tobacco. Tob Control.

[B75] Hussain SF, Tjeder-Burton S, Campbell IA, Davies PD (1993). Attitudes to smoking and smoking habits among hospital staff. Thorax.

[B76] Yaacob I, Abdullah ZA (1993). Smoking habits and attitudes among doctors in a Malaysian hospital. Southeast Asian J Trop Med Public Health.

[B77] Doll R, Peto R, Wheatley K, Gray R, Sutherland I (1994). Mortality in relation to smoking: 40 years' observations on male British doctors. BMJ.

[B78] Kaetsu A, Fukushima T, Moriyama M, Shigematsu T (2002). Change of the smoking behavior and related lifestyle variables among physicians in Fukuoka, Japan: A longitudinal study. J Epidemiol.

[B79] Jormanainen VJ, Myllykangas MT, Nissinen A (1997). Decreasing the prevalence of smoking among Finnish physicians. Eur J Public Health.

[B80] Brink SG, Gottlieb NH, McLeroy KR, Wisotzky M, Burdine JN (1994). A community view of smoking cessation counselling in the practices of physicians and dentists. Public Health Rep.

[B81] Hensrud DD, Sprafka JM (1993). The smoking habits of Minnesota physicians. Am J Pub Health.

[B82] Waalkens HJ, Cohen Schotanus J, Adriaanse H, Knol K (1992). Smoking habits in medical students and physicians in Groningen, The Netherlands. Eur Respir J.

[B83] Kawane H (2001). Smoking among Japanese physicians. JAMA.

[B84] Kawane H (1993). The prevalence of smoking among physicians in Japan. Am J Public Health.

[B85] Kawane H (1991). Smoking among older chest physicians. Chest.

[B86] Dekker HM, Looman CWN, Adriaanse HP, Van Der Maas PJ (1993). Prevalence of smoking in physicians and medical students, and the generation effect in the Netherlands. Soc Sci Med.

[B87] Hughes PH, Brandenburg N, Baldwin DC, Storr CL, Williams KM, Anthony JC (1992). Prevalence of substance use among US physicians. JAMA.

[B88] Scott HD, Tierney JT, Buechner JS, Waters WJ (1992). Smoking rates among Rhode Island physicians: Achieving a smoke-free society. Am J Prev Med.

[B89] Fowler G, Mant D, Fuller A, Jones L (1989). The "Help Your Patient Stop" initiative. Evaluation of smoking prevalence and dissemination of WHO/UICC guidelines in UK general practice. Lancet.

[B90] Saeed AA (1991). Attitudes and behaviour of physicians towards smoking in Riyadh city, Saudi Arabia. Trop Geogr Med.

[B91] Nutbeam D, Catford J (1990). Modifiable risks for cardiovascular disease among general practitioners in Wales. Public Health.

[B92] Hughes PH, Baldwin DC, Sheehan DV, Conard S, Storr CL (1992). Resident physician substance use, by specialty. Am J Psychiatry.

[B93] Davies PD, Rajan K (1989). Attitudes to smoking and smoking habit among the staff of a hospital. Thorax.

[B94] Cheng KK, Lam TH (1990). Smoking among young doctors in Hong Kong: A message to medical educators. Med Educ.

[B95] Stillman FA, Becker DM, Swank RT, Hantula D, Moses H, Glantz S (1990). Ending smoking at the Johns Hopkins Medical Institutions. An evaluation of smoking prevalence and indoor air pollution. JAMA.

[B96] Sarkar D, Dhand R, Malhotra A, Malhotra S, Sharma BK (1990). Perceptions and attitude towards tobacco smoking among doctors in Chandigarh. Indian J Chest Dis Allied Sci.

[B97] Franceschi S, Serraino D, Talamini R, Candiani E (1986). Personal habits and attitudes towards smoking in a sample of physicians from the north-east of Italy. Int J Epidemiol.

[B98] Linn LS, Yager J, Cope D, Leake B (1986). Health habits and coping behaviors among practicing physicians. West J Med.

[B99] Joossens L, Demedts M, Prignot J, Bartsch P, Gyselen A (1987). Smoking habits of Belgian physicians: Effects of consonancy behaviour and of age. Acta Clin Belg.

[B100] Kaetsu A, Fukushima T, Moriyama M, Shigematsu T (2002). Smoking behavior and related lifestyle variables among physicians in Fukuoka, Japan: A cross sectional study. J Epidemiol.

[B101] Sachs DP (1983). Smoking habits of pulmonary physicians. N Engl J Med.

[B102] Sachs DP (1984). Treatment of cigarette dependency. What American pulmonary physicians do. Am Rev Respir Dis.

[B103] Seiler ER (1983). Smoking habits of doctors and their spouses in south east Scotland. J R Coll Gen Pract.

[B104] Senior SL (1982). Study of smoking habits in hospital and attitudes of medical staff towards smoking. CMAJ.

[B105] Fortmann SP, Sallis JF, Magnus PM, Farquhar JW (1985). Attitudes and practices of physicians regarding hypertension and smoking: The Stanford Five City Project. Prev Med.

[B106] Hay DR (1984). Intercensal trends in cigarette smoking by New Zealand doctors and nurses. NZ Med J.

[B107] Ballal SG (1984). Cigarette smoking and respiratory symptoms among Sudanese doctors. East Afr Med J.

[B108] Wyshak G, Lamb GA, Lawrence RS, Curran WJ (1980). A profile of the health-promoting behaviors of physicians and lawyers. N Engl J Med.

[B109] Wells KB, Lewis CE, Leake B, Ware JE (1984). Do physicians preach what they practice? A study of physicians' health habits and counseling practices. JAMA.

[B110] Dodds AM, Rankin DW, Hill DJ, Gray NJ (1979). Attitudes and smoking habits of doctors in Victoria. Comm Health Stud.

[B111] Hay DR (1980). Cigarette smoking by New Zealand doctors: Results from the 1976 population census. NZ Med J.

[B112] Aaro LE, Bjartveit K, Vellar OD, Berglund EL (1977). Smoking habits among Norwegian doctors 1974. Scand J Soc Med.

[B113] Rankin DW, Gray NJ, Hill DJ, Evans DR (1975). Attitudes and smoking habits of Australian doctors. Med J Aust.

[B114] Christmas BW, Hay DR (1976). The smoking habits of New Zealand doctors: A review after ten years. NZ Med J.

[B115] Audet B (1994). When it comes to smoking, Japanese MDs do not set a good example for their patients. CMAJ.

[B116] Bartscherer DJ, Reichert VC, Folan P, Degaetano C, Jacobsen DR, Miceli L (2005). Tobacco and the health care industry. Clin Occup Environ Med.

[B117] Hay DR, Christmas BW (1976). The smoking habits of women doctors and doctors' wives in New Zealand. Prev Med.

[B118] Adriaanse H, van Reek J (1989). Physicians' smoking and its exemplary effect. Scand J Prim Health Care.

[B119] Anon (1995). Smoking and health: A physician's responsibility. A statement of the joint committee on smoking and health. American College of Chest Physicians, American Thoracic Society, Asia Pacific Society of Respirology, Canadian Thoracic Society, European Respiratory Society, International Union Against Tuberculosis and Lung Disease. Eur Respir J.

[B120] Chapman S (1995). Doctors who smoke. BMJ.

[B121] Centers for Disease Control and Prevention (CDC) (1993). Smoking control among health-care workers – World No-Tobacco Day, 1993. MMWR Morb Mortal Wkly Rep.

[B122] Clever LH, Arsham GM (1984). Physicians' own health – some advice for the advisors. West J Med.

[B123] Fiore MC, Bailey WC, Cohen SJ, Dorfman SF, Goldstein MG, Gritz ER (2000). Treating Tobacco Use and Dependence. Quick Reference Guide for Clinicians.

[B124] Raw M, McNeill A, West R (1998). Smoking cessation guidelines for health professionals. A guide to effective smoking cessation interventions for the health care system. Health Education Authority. Thorax.

[B125] Zwar N, Richmond R, Borland R, Stillman S, Cunningham M, Litt J (2005). Smoking cessation guidelines for Australian general practice. Aust Fam Physician.

[B126] Rahman M, Fukui T (2003). Biomedical publication – global profile and trend. Public Health.

[B127] Smith DR, Leggat PA (2006). A comparison of tobacco smoking among dentists in 15 countries. Int Dent J.

